# Supplementary material for: The contribution of electrostatics to hydrogen exchange in the unfolded protein state
Source: Biophys J. 2021 Aug 8;120(18):4107–14. doi: 10.1016/j.bpj.2021.08.003 (PMC8510857; doi:10.1016/j.bpj.2021.08.003)
Supplement: Document S1. Figs. S1–S2 and Tables S1–S2 [file mmc1.pdf]

Biophysical Journal, Volume *120*

Supplemental information

The contribution of electrostatics to hydrogen exchange in the unfolded protein state

Rupashree Dass, Enrico Corlianò, and Frans A.A. Mulder

Supporting Material to:

The contribution of electrostatics to hydrogen exchange in the unfolded protein state

Rupashree Dass[1,2], Enrico Corlianò[3] and Frans A. A. Mulder[1,2*]

[1,2] Department of Chemistry and Interdisciplinary Nanoscience Center (iNANO), Aarhus University 8000, Aarhus, Denmark; [3] Department of Chemistry, University of Florence, 50019, Sesto Fiorentino, (FI) - Italy

Table S1. Experimental and computed parameters for a-synuclein: Measured hydrogen exchange rate, protection factor, amide hydrogen temperature coefficient, signal intensity ratio in the presence of negative divided by neutral PRE agent, calculated electrostatic potential, and calculated protection factor.

peptide bond	rate (s^{-1})	measured PF	Temp. coeff. (ppb/K)	I_{neg}/I_{neu}	electrostatic potential (mV)	expected PF
D2N-M1C	-	-	-	-	-3.214	1.133
V3N-D2C	-	-	-7.222	0.992	-0.573	1.023
F4N-V3C	206.017	1.364	-6.657	-	2.465	0.908
M5N-F4C	210.233	3.596	-5.043	0.879	5.587	0.804
K6N-M5C	230.763	3.432	-6.659	0.926	8.497	0.718
G7N-K6C	-	-	-5.689	-	8.043	0.731
L8N-G7C	1084.645	0.242	-5.819	0.828	7.817	0.738
S9N-L8C	1617.746	0.602	-5.992	-	9.099	0.702
K10N-S9C	-	-	-6.968	-	10.774	0.657
A11N-K10C	-	-	-7.395	-	9.640	0.687
K12N-A11C	1237.161	0.497	-6.965	-	7.795	0.738
E13N-K12C	491.806	0.559	-	-	3.393	0.876
G14N-E13C	609.032	1.460	-5.903	-	2.714	0.900
V15N-G14C	294.599	0.675	-5.751	1.014	3.011	0.889
V16N-V15C	-		-8.024	-	2.992	0.890

A17N-V16C	-	-	-7.000	-	2.895	0.893
A18N-A17C	714.763	0.943	-6.835	-	2.715	0.900
A19N-A18C	772.548	0.872	-6.227	-	2.204	0.918
E20N-A19C	410.93	0.506	-4.838	0.974	2.641	0.902
K21N-E20C	744.21	0.585	-6.151	0.908	6.759	0.769
T22N-K21C	885.606	0.854	-6.805	-	8.248	0.725
K23N-T22C	-		-6.724	-	8.913	0.707
Q24N-K23C	959.216	1.063	-	-	6.568	0.774
G25N-Q24C	-	-	-4.395	-	4.232	0.848
V26N-G25C	275.064	0.723	-5.941	0.972	2.471	0.908
A27N-V26C	464.756	1.050	-5.505	1.066	0.401	0.985
E28N-A27C	521.521	0.399	-5.959	0.932	-1.323	1.053
A29N-E28C	431.223	1.106	-5.732	0.962	0.669	0.974
A30N-A29C	805.425	0.837	-5.327		3.129	0.885
G31N-A30C	1251.47	1.007	-4.254	0.894	5.729	0.800
K32N-G31C	1104.19	0.823	-3.568	0.881	8.199	0.727
T33N-K32C	-	-	-6.262	0.906	7.603	0.744
K34N-T33C	-	-	-7.016	0.972	6.160	0.787
E35N-K34C	202.185	1.360	-	-	2.086	0.922
G36N-E35C	-	-	-5.622	-	1.705	0.936
V37N-G36C	-	-	-6.203	0.919	2.306	0.914
L38N-V37C	187.449	0.683	-6.765	0.957	2.639	0.902
T39N-L38C	1317.285	0.169	-7.943	-	3.006	0.890
V40N-T39C	-	-	-5.068	0.921	3.542	0.871
G41N-V40C	1177.67	0.772	-1.986	0.843	4.425	0.842
S42N-G41C	-	-	-6.165	-	6.129	0.788
K43N-S42C	-	-	-6.178	-	7.962	0.733
T44N-K43C	1175.190	0.643	-6.111	0.855	6.849	0.766
K45N-T44C	-	-	-7.019	-	4.945	0.825
E46N-K45C	-	-	-	-	0.431	0.983

G47N-E46C	513.403	1.735	-5.708	-	-0.396	1.016
V48N-G47C	163.944	1.214	-5.986	0.907	-0.275	1.011
V49N-V48C	140.685	0.692	-7.265	0.928	-0.486	1.019
H50N-V49C	367.473	1.061	-9.486	0.958	-0.771	1.030
G51N-H50C	885.131	1.966	-6.219	0.916	-1.076	1.043
V52N-G51C	274.331	0.725	-7.068	0.912	-1.390	1.056
T53N-V52C	-	-	-6.865	0.954	-1.718	1.069
T54N-T53C	814.16	0.705	-8.041	0.936	-2.086	1.085
V55N-T54C	-	-	-8.595		-2.567	1.105
A56N-V55C	672.044	0726	-7.211	0.924	-3.441	1.143
E57N-A56C	374.917	0.555	-6.535	1.013	-3.466	1.145
K58N-E57C	629.158	0.691	-6.978	0.984	0.029	0.999
T59N-K58C	1019.68	0.741	-7.327	0.946	0.602	0.977
K60N-T59C	-	-	-6.965	0.924	-0.367	1.014
E61N-K60C	-	-	-	-	-4.261	1.181
Q62N-E61N	-	-	-	-	-4.637	1.198
V63N-Q62C	368.242	0.578	-7.443	1.003	-4.169	1.176
T64N-V63C	1240.27	0.335	-6.711	0.939	-4.099	1.173
N65N-T64C	-	-	-6.138	-	-4.149	1.175
V66N-N65C	749.675	0.374	-7.554	0.912	-4.246	1.180
G67N-V66C	1047.51	0.867	-6.768	0.826	-4.364	1.185
G68N-G67C	953.059	1.951	-5.397	0.893	-4.491	1.191
A69N-G68C	961.042	1.037	-5.462	0.912	-4.619	1.197
V70N-A69C	-	-	-7.941	-	-4.746	1.203
V71N-V70C	-	-	-8.368	0.929	-4.866	1.209
T72N-V71C	1173.68	0354	-7.319	0.918	-4.976	1.214
G73N-T72C	1241.47	1.602	-6.303	0.908	-5.068	1.218
V74N-G73C	-	-	-5.968	0.929	-5.135	1.221
T75N-V74C	1018.64	0.408	-7.354	0.916	-5.162	1.223
A76N-T75C	1103.78	0.969	-7.649	0.889	-5.123	1.221

V77N-A76C	-	-	-9.000	0.908	-4.966	1.213
A78N-V77C	889.721	0.548	-7.746	-	-4.567	1.195
Q79N-A78C	197.894	3.911	-8.581	-	-3.522	1.147
K80N-Q79C	304.110	3.202	-7.657	0.956	-2.671	1.110
T81N-K80C	1016.72	0.743	-7.686	0.940	-5.525	1.240
V82N-T81C	296.235	0.719	-7.862	0.991	-9.155	1.428
E83N-V82C	298.986	0.505	-6.862	1.022	-12.010	1.596
G84N-E83C	1011.41	0.878	-7.319	1.097	-11.158	1.544
A85N-G84C	1078.95	0.924	-5.989	0.949	-10.111	1.483
G86N-A85C	-	-	-6.349	0.949	-9.704	1.459
S87N-G86C	1280.53	1.827	-4.370	0.963	-9.532	1.449
I88N-S87C	552.73	0.452	-6.454	1.110	-9.463	1.446
A89N-I88C	633.313	0.626	-7.041	0.938	-9.438	1.444
A90N-A89C	1009.65	0.667	-7.805	0.936	-9.416	1.443
A91N-A90C	808.213	0.833	-6.905	-	-9.362	1.440
T92N-A91C	1105.83	0.519	-7.681	0.974	-9.225	1.432
G93N-T92C	1361.04	1.462	-5.470	0.927	-8.923	1.416
F94N-G93C	829.572	0.691	-5.365	-	-8.282	1.381
V95N-F94C	90.5266	1.701	-5.324	0.960	-6.783	1.302
K96N-V95C	483.068	0.921	-6.105	0.996	-4.906	1.211
K97N-K96C	1189.72	0.681	-6.446	-	-7.229	1.325
D98N-K97C	933.156	0.476	-5.327	1.207	-12.969	1.657
Q99N-D98C	633.412	0.806	-6.157	-	-14.589	1.765
L100N-Q99C	1143.34	0.245	-6.978	0.974	-15.040	1.796
G101N-L100C	463.91	1.668	-6.068	1.128	-15.347	1.818
K102N-G101C	468.09	1.941	-6.008	-	-16.279	1.885
N103N-K102C	1197.08	2.297	-6.268	1.147	-21.757	2.333
E104N-N103C	459.658	0.946	-4.900	1.140	-28.088	2.986
E105N-E104C	-	-	-	-	-30.388	3.265
G106N-E105C	-	-	-5.811	1.128	-29.144	3.111

A107N-G106C	147.667	6.751	-5.459	1.108	-28.732	3.062
P108N-A107C	36.4415	-	-		-29.728	3.183
Q109N-P108C	202.128	2.201	-7.481	1.121	-32.066	3.486
E110N-Q109C	-	-	-7.132	1.187	-34.850	3.885
G111N-E110C	385.028	2.308	-5.576	1.257	-34.741	3.869
I112N-G111C	-	-	-6.911	1.104	-35.290	3.952
L113N-I112C	34.818	2.986	-7.443	-	-37.854	4.367
E114N-L113C	80.865	1.582	-6.665	-	-41.680	5.069
D115N-E114C	77.02	3.103	-6.073	1.261	-42.530	5.240
M116N-D115C	48.674	8.937	-7.281	-	-40.432	4.829
P117N-M116C	48.962	-	-	-	-39.690	4.691
V118N-P117C	5.903	13.110	-9.303	1.148	-41.151	4.966
D119N-V118C	65.837	3.721	-8.484	1.252	-43.637	5.471
P120N-D119C	0.452	-	-	-	-44.085	5.567
D121N-P120C	70.124	2.766	-4.665	1.311	-45.156	5.804
N122N-D121C	185.90	7.423	-3.368	1.284	-44.682	5.698
E123N-N122C	147.773	2.944	-5.562	-	-44.866	5.739
A124N-E123C	82.776	5.762	-6.195	1.321	-43.117	5.361
T125N-A124C	70.93	5.103	-6.581	-	-42.762	5.287
E126N-T125C	106.14	2.204	-2.941	1.392	-43.672	5.478
M127N-E126C	-	-	-6.724	1.293	-42.061	5.145
P128N-M127C	-	-	-	-	-41.322	4.999
S129N-P128C	232.832	3.904	-7.565	1.417	-42.737	5.282
E130N-S129C	-	-	-6.497	1.371	-45.510	5.885
E131N-E130C	-	-	-5.765	-	-45.374	5.853
G132N-E131N	-	-	-6.108	-	-42.336	5.200
T133N-G132C	150.157	3.562	-5.484	1.210	-40.685	4.876
Q134N-T133C	192.421	4.510	-4.189	-	-41.255	4.986
D135N-Q134C	154.626	3.466	-4.846	1.541	-42.857	5.307
T136N-D135C	39.900	5.989	-5.995	-	-42.421	5.218

E137N-T136C	56.805	4.119	-5.822	-	-42.608	5.256
P138N-E137C	-	-	-	-	-41.261	4.987
E139N-P138C	56.491	2.124	-6.430	1.266	-40.639	4.868
A140N-E139C	19.876	0.38	-8.170	1.133	-38.414	4.464

Table S2 Protection factors before (PF) and after (PF*) taking electrostatic contributions in the unfolded state into account. The PF* data are plotted in Figure 4.

Peptide bond	Protection factors from Nishimura et. al.[a]	Data for Fig4. Plot a	Data for Fig4. Plot b
D2N-M1C	-	-	-
V3N-D2C	0.31677	0.357	-
F4N-V3C	0.62459	0.739	1.790
M5N-F4C	18.002	15.404	3.958
K6N-M5C	5.3867	5.338	4.266
G7N-K6C	6.2648	4.134	-
L8N-G7C	0.51799	0.363	0.246
S9N-L8C	1.3924	1.546	0.816
K10N-S9C	1.0597	1.247	-
A11N-K10C	13.256	9.769	-
K12N-A11C	9.7554	7.358	0.539
E13N-K12C	0.82597	0.529	0.528
G14N-E13C	1.748	1.699	1.779
V15N-G14C	3.5179	4.875	1.008
V16N-V15C	-	-	-
A17N-V16C	1.4884	1.301	-
A18N-A17C	1.3019	1.130	1.056
A19N-A18C	2.035	1.748	0.970
E20N-A19C	0.38764	0.325	0.552
K21N-E20C	1.2679	1.867	0.929
T22N-K21C	0.73522	0.701	1.040
K23N-T22C	2.4053	1.658	-
Q24N-K23C	7.8499	5.149	1.049
G25N-Q24C	3.1045	1.970	-
V26N-G25C	0.81468	0.738	0.853
A27N-V26C	5.1965	4.319	1.156
E28N-A27C	0.76794	0.579	0.405
A29N-E28C	6.2644	7.489	1.506
A30N-A29C	3.6938	4.463	1.153
G31N-A30C	1.0849	0.917	1.137
K32N-G31C	-	-	1.029

T33N-K32C	0.74768	0.460	-
K34N-T33C	0.93317	0.602	-
E35N-K34C	2.9098	1.620	1.206
G36N-E35C	-	-	-
V37N-G36C	1.1695	1.446	-
L38N-V37C	1.1899	0.943	0.747
T39N-L38C	1.2726	1.019	0.188
V40N-T39C	0.51294	0.416	-
G41N-V40C	0.97547	0.808	0.886
S42N-G41C	2.3821	2.044	-
K43N-S42C	0.44063	0.406	-
T44N-K43C	0.72346	0.418	0.612
K45N-T44C	-	-	-
E46N-K45C	-	-	-
G47N-E46C	-	-	1.881
V48N-G47C	1.5768	1.655	1.604
V49N-V48C	1.1168	0.732	0.685
H50N-V49C	3.0732	1.978	1.041
G51N-H50C	1.6836	1.061	1.908
V52N-G51C	0.66464	0.410	0.696
T53N-V52C	0.49958	0.301	-
T54N-T53C	0.49605	0.292	0.659
V55N-T54C	0.79268	0.456	-
A56N-V55C	1.3951	0.779	0.657
E57N-A56C	1.3162	0.701	0.485
K58N-E57C	2.4883	2.265	0.866
T59N-K58C	0.71568	0.407	0.695
K60N-T59C	2.2053	0.866	-
E61N-K60C	2.1486	0.742	-
Q62N-E61N	-	-	-
V63N-Q62C	-	-	0.648
T64N-V63C	0.36389	0.177	0.285
N65N-T64C	-	-	-
V66N-N65C	0.64729	0.311	0.319
G67N-V66C	0.85825	0.407	0.736
G68N-G67C	1.924	0.901	1.647
A69N-G68C	-	-	0.871
V70N-A69C	0.99733	0.455	-
V71N-V70C	0.70609	0.318	-
T72N-V71C	0.56829	0.252	0.293
G73N-T72C	1.3899	0.609	1.321
V74N-G73C	1.2691	0.549	-
T75N-V74C	0.45245	0.194	0.334
A76N-T75C	0.80088	0.340	0.793

V77N-A76C	0.88744	0.374	-
A78N-V77C	1.3062	0.550	0.452
Q79N-A78C	1.2575	0.534	3.274
K80N-Q79C	1.3276	0.585	2.792
T81N-K80C	0.98905	0.260	0.467
V82N-T81C	-	-	0.432
E83N-V82C	0.44983	0.148	0.354
G84N-E83C	0.78135	0.385	0.789
A85N-G84C	1.0257	0.481	0.803
G86N-A85C	0.85092	0.260	-
S87N-G86C	1.6057	0.494	1.252
I88N-S87C	0.86683	0.266	0.312
A89N-I88C	1.2977	0.396	0.434
A90N-A89C	1.9714	0.597	0.462
A91N-A90C	2.4619	0.738	0.578
T92N-A91C	0.46124	0.137	0.360
G93N-T92C	0.98036	0.291	1.021
F94N-G93C	5.8833	1.749	0.489
V95N-F94C	0.74357	0.225	1.232
K96N-V95C	1.5722	0.502	0.707
K97N-K96C	5.3815	1.070	0.393
D98N-K97C	2.3677	0.261	0.187
Q99N-D98C	1.3114	0.325	0.520
L100N-Q99C	1.1776	0.393	0.187
G101N-L100C	1.6718	0.336	0.929
K102N-G101C	7.5546	1.463	1.068
N103N-K102C	2.7273	0.287	0.850
E104N-N103C	1.4365	0.125	0.302
E105N-E104C	1.0612	0.186	-
G106N-E105C	1.231	0.303	-
A107N-G106C	6.3504	0.939	2.913
P108N-A107C	-	-	-
Q109N-P108C	1.4064	0.122	0.692
E110N-Q109C	1.6737	0.129	-
G111N-E110C	1.9667	0.225	0.852
I112N-G111C	5.5501	0.581	-
L113N-I112C	3.711	0.234	0.756
E114N-L113C	2.8423	0.157	0.362
D115N-E114C	2.1891	0.172	0.878
M116N-D115C	3.1061	0.370	3.282
P117N-M116C	-	-	-
V118N-P117C	9.596	0.463	-
D119N-V118C	4.463	0.200	0.749
P120N-D119C	-	-	-

D121N-P120C	5.4173	0.333	0.667
N122N-D121C	7.3308	0.460	1.834
E123N-N122C	-	-	0.694
A124N-E123C	10.876	0.686	1.440
T125N-A124C	7.62	0.480	1.278
E126N-T125C	4.6555	0.187	0.417
M127N-E126C	7.1394	0.473	-
P128N-M127C	-	-	-
S129N-P128C	2.8413	0.122	0.781
E130N-S129C	0.91605	0.037	-
E131N-E130C	0.95148	0.058	-
G132N-E131N	2.9857	0.294	-
T133N-G132C	4.1834	0.276	0.920
Q134N-T133C	2.5043	0.112	0.925
D135N-Q134C	8.061	0.355	0.695
T136N-D135C	8.5766	0.609	1.618
E137N-T136C	5.5458	0.375	1.060
P138N-E137C	-	-	-
E139N-P138C	3.0215	0.220	0.572
A140N-E139C	2.9058	0.238	0.112

[a] taken from Ref 33 of the main text.

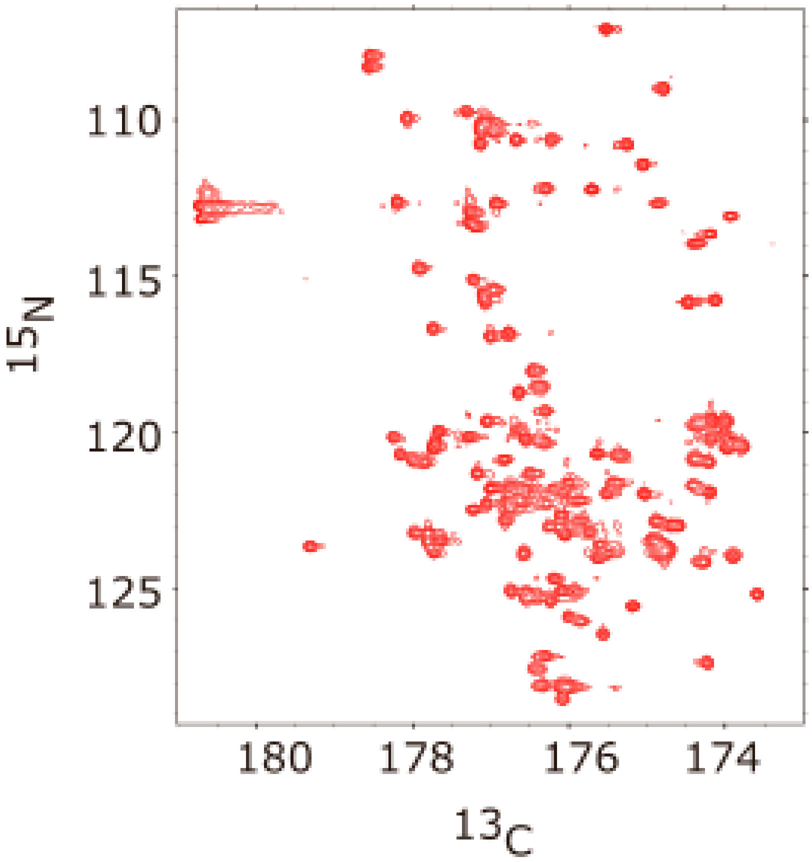

Figure S1. 2D CON spectrum for the measurement of hydrogen exchange for α-synuclein at 298K and pH 9.

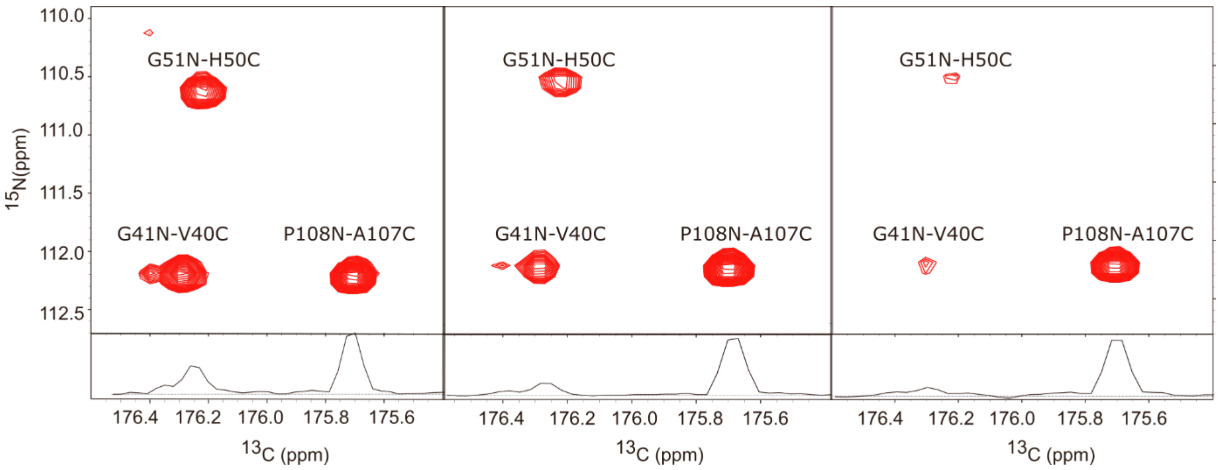

Figure S2. Region of the 2D CON spectrum for the measurement of hydrogen exchange obtained for reference experiment (left), with ncpmg=8 (middle) and ncpmg=2 (right). The correlation due to Pro108 is aliased in the ^{15}N dimension.
